# Review of the Mechanical Behavior of Different Implant–Abutment Connections

**DOI:** 10.3390/ijerph17228685

**Published:** 2020-11-23

**Authors:** Ana Sofia Vinhas, Carlos Aroso, Filomena Salazar, Paula López-Jarana, José Vicente Ríos-Santos, Mariano Herrero-Climent

**Affiliations:** 1 Department of Periodontology, Instituto Universitário de Ciências da Saúde, 4585-116 Gandra, Portugal; ana.vinhas@iucs.cespu.pt (A.S.V.); filomena.salazar@cespu.pt (F.S.); plopezjarana@hotmail.com (P.L.-J.); 2 Department of Prosthodontics, Instituto Universitário de Ciências da Saúde, 4585-116 Gandra, Portugal; carlos.ribeiro@iucs.cespu.pt; 3 Department of Periodontology, School of Dentistry, Universidad de Sevilla, C/Avicena S/N, 41009 Sevilla, Spain; 4 Porto Dental Institute, 4150-518 Porto, Portugal; dr.herrero@herrerocliment.com

**Keywords:** implant–abutment connection, preload, tightening torque, cyclic loading, misfit, microleakage

## Abstract

*Introduction:* Different implant–abutment connections have been developed to reduce mechanical and biological failure. The most frequent complications are loss of preload, screw loosening, abutment or implant fracture, deformations at the different interfaces, and bacterial microleakage. Aim: To review the evidence indicating whether the implant–abutment connection type is significant regarding the following issues: (1) maintenance of the preload in static and dynamic in vitro studies; (2) assessment of possible deformations at the implant–abutment interfaces, after repeated application of the tightening torque; (3) evaluation of the sealing capability of different implant connections against microleakage. *Materials and Methods:* In June 2020, an electronic literature search was performed in Medline, EBSCO host, and PubMed databases. The search was focused on the ability of different implant connections to maintain preload, resist deformation after tightening and retightening, and prevent microleakage. The related titles and abstracts available in English were screened, and the articles that fulfilled the inclusion criteria were selected for full-text reading. *Results:* The literature search conducted for this review initially resulted in 68 articles, among which 19 articles and 1 systematic review fulfilled the criteria for inclusion. The studies were divided according to the three proposed objectives, with some studies falling into more than one category (maintenance of preload, surface abutment–implant deformation, and resistance to microleakage). *Conclusions:* Conical abutment appears to result in fewer mechanical complications, such as screw loosening or fractures, and higher torque preservation. After SEM evaluation, damage was observed in the threads of the abutment screws, before and after loading in internal and external connections. Internal hexagon implants and predominantly internal conical (Morse taper) implants showed less microleakage in dynamic loading conditions. We suggest further studies to guarantee excellence in methodological quality.

## 1. Introduction

In recent years, geometries of implant connections have been developed with different mechanical, biological, and esthetic characteristics. Two basic geometries are available: internal and external connections. External connections usually have an external hexagon on the implant platform, whereas internal connections can be divided into internal hexagons, internal octagons, and Morse taper connections [1]. The osseointegrated implant and the prosthetic abutment are joined by a screw and have, therefore, been named a screw joint [2].

The external hexagon was the first connection system adopted in modern implantology by Branemark [3], based on the existence of a hexagon (0.7 mm height); however, this connection has been extensively modified in terms of diameter, height, and insertion torque. This kind of connection presents some advantages. First, it is adequate for the two-step surgical procedure preferred by Branemark [3] because it alleviates the second stage and the connection phase with the healing abutment. Second, it simplifies the recording of the external connection in the impression and the prosthetic phase due to its adjustability and compatibility with different prosthetic solutions. However, it also presents a number of disadvantages, such as little contact length between the restoration and the hexagonal part of the implant head, some degree of rotation between the platform and the internal hexagon of the restoration, and high tension created in the screw connection. It has been speculated that under high occlusal loads, the external hexagon might allow for micromovements of the abutment, causing instability of the joint, which may result in abutment screw loosening or even fatigue fractures [4,5,6]. The literature has shown the loosening rate of this type of connection to be between 6% and 48% [7], thus presenting a mechanical difficulty for the maintenance of the preload (torque for the removal of the pillar must be 10% lower than that of installation) [8,9].

Internal connections have been introduced with a Morse cone of different degrees of inclination, depending on the commercial brand [10], to lower or eliminate the mechanical complications of the external connection and to reduce the stress transferred to the crestal bone [11,12,13,14]. In the internal hexagonal system, the hexagon and the screw pass into the implant body so the prosthetic component is more stable. The internal hexagon connection was developed as an evolution of the external hexagon, with the aim of increasing the load absorption under a lateral force. This reduces mechanical and biological complications, such as screw loosening, fracture, and marginal bone loss. The greater depth of the connection in the fixture body allows more homogeneous dissipation of the mechanical stress; the stress is spread on the implant wall and, consequently, to the bone surrounding the entire implant and not only at the crestal level. [15] A conical connection is a particular kind of internal connection in which the abutment is fixed to the implant using the mechanical properties of a machine taper. A male member of a conical shape fits into a female socket, which has a matching taper of equal angle. The connection works by locking the two components by mechanical friction between the wall of the abutment and the implant. Although mechanical friction has been demonstrated to be strong enough, implant companies have also implemented screw retention and antirotational systems. However, to date, no qualitative data exists comparing the mechanical behavior of external and internal connections [7]. The internal connection using a Morse cone creates a more accurate bond between the implant and the abutment, which reduces the movement of the interface and decreases the loosening of the screw (torque for the removal of the abutment must be 17% greater than that of installation) [8,9]. The Morse cone has an internal cone of 8° or 11°, which can protect against screw loosening [9,16].

As described previously, the different implant–abutment connection designs have very different characteristics, which can affect mechanical stability. The failure of an implant is related to two problems: biological and mechanical factors. Biological causes essentially relate to periimplantitis, which affects the soft and hard tissues around the implants, whereas mechanical causes involve prosthetic components, namely, overload of the prothesis–implant–pillar complex, implant fracture, abutment fracture, loosening of the screws, and fracture of the superstructure (metal/ceramic) [17,18].

Abutment screw stability can be affected by preload, the effect of settling, and screw geometry [2,19,20]. Preload is the force that is generated when the screw is tightened using a given torque [2,19,21]. Torque is defined as the movement produced by applying tangential force to the screw and is usually expressed in newton centimeters (Ncm). When applying the preload to a screw, the connected elements are kept in compression, and the screw receives small impacts because most of the load is absorbed by the components of the implant–abutment junction [22]. The initial preload on the screw is usually inserted by applying torque using a torque wrench. One of the major causes of screw loosening is the “loss of preload”. Only 10% of the initial torque is transformed into preload, whereas the remaining 90% is used to overcome the friction between the surface irregularities [2,19,21]. In the tightening of the components of the connection, tension occurs with a consequent compression between the structures of the joint. Screw loosening is one of the most common mechanical complications of implant treatment, with an estimated annual rate of 2.1% [23]. Estimated rates are 10.4% and 20.8% over 5 and 10 years, respectively [24]. From a clinical perspective, the loosening of the screw is greater in external connections than in internal connections, with an incidence of loose screws of 38% in external hexagon systems [25,26]. However, the ratio of torque to preload is not linear and is affected by several factors: coefficient of friction, geometry, and properties of contact surface materials. The first is the most influential and depends on the hardness of the threads, the finish of the surfaces, the lubricant used, and the tightening speed [27]. A preload torque between 10 and 35 Ncm is recommended by different manufacturers, depending on the screw manufacturing material and the morphology of the abutment–implant connection [2].

Another important phenomenon experienced by the screw joint is the settling effect. This occurs because neither the interior torque nor the screw is perfectly fabricated and without irregularity; therefore, these rough areas are smoothed, causing a loss of 2–10% of the initial preload [2,19]. Torque loosening causes micromovements in the interface screw, abutment, and implant body, which cause both mechanical and biological problems. This misalignment of the fitting results in the colonization of bacteria at the interface and is a major challenge to the success of the implant. Microleakage may be defined as the clinically undetectable passage of bacteria, fluids, molecules, or ions between a cavity wall and the restorative materials. Microleakage depends directly on the marginal accuracy of the components (fit, tolerance, presence of microgaps) [28]. The problems associated with microgaps between the implant and the abutment are both biological and mechanical. The biological problem relates to the presence of bacteria that have been found in the apical portion of the abutment screw; in vivo, this could produce a bacterial reservoir that could interfere with the long-term health of the peri-implant tissues. The mechanical problem relates to the micromovements and possible loosening or fracture due to the fatigue of screw-retained abutments [29]. The external hexagon, in fact, is subject to micromovements under lateral load, and this may create a microgap at the abutment–fixture interface [30]. Different authors have investigated this condition, and this microgap can lead to microleakage and bacterial infiltration that may affect the long-term success of dental implants [31].

The stability and integrity of the abutment–implant connection, by means of a screw, are fallible from the moment the prosthetic elements are attached. This fallibility depends on the applied preload, wear of the components, and function. It is necessary to evaluate and quantify, with in vitro studies, the loss of torque before and after loading and the integrity of the system structures in the different connections. The current work aims to review the existing literature to evaluate, according to the type of connection, the maintenance of the preload, the assessment of possible deformations at the different interfaces after repeated application of the tightening torque, and the sealing capability of different implant connections against microleakage.

## 2. Materials and Methods

### 2.1. Search Strategy and Study Selection

The following analysis was performed according to the guidelines and the principles of an integrative review. The review is focused on the guiding question, “is the implant–abutment connection design important in the mechanical behavior of dental implants?”. Dental literature in Medline, EBSCO Host, and PubMed databases was searched from January 2004 to June 2020. The literature search was limited to journals available in English. The keywords were free-text words and included a combination of the following: implant abutment connections; preload; tightening torque; cyclic loading; implant abutment deformation; misfit; microleakage. Manual and electronic searches were performed to select the relevant articles.

### 2.2. Inclusion and Exclusion Criteria

**Figure 1:**
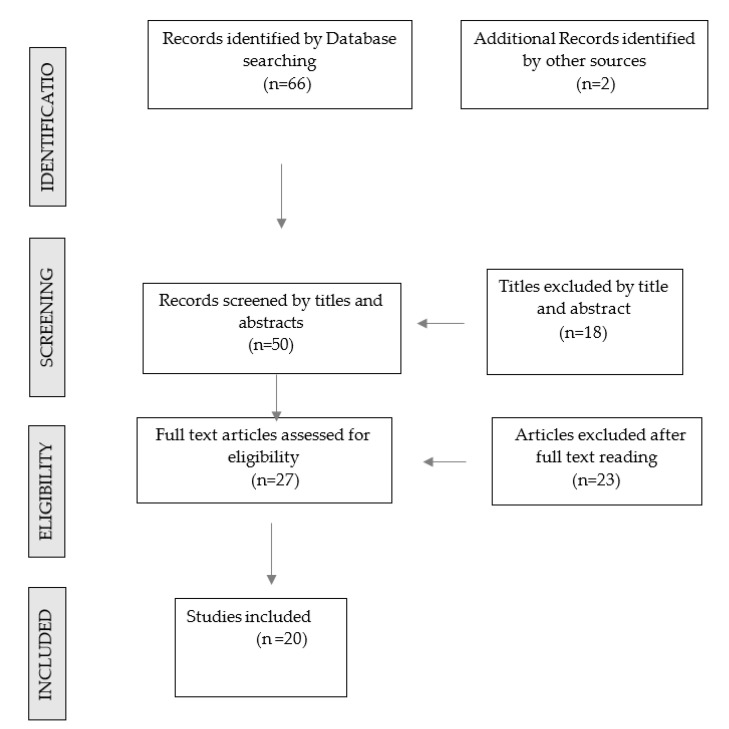
Flow chart presenting the screening of articles related to the mechanical behavior of different implant connections to be included in the review.

The screening of the articles was conducted, as shown in the flow chart of Figure 1. In the present review, the following inclusion criteria were as follows: articles in English published between January 2004 and June 2020; in vitro studies and systematic reviews, with a clear aim of investigating the relationship of different implant connections to loss of preload, surface abutment–implant deformation, and resistance to microleakage. Case reports, human trials, and studies involving animals were excluded.

### 2.3. Data Extraction

All studies meeting the criteria were obtained, screened independently, and analyzed according to the stages of an integrative review process. The literature search initially resulted in a total of 68 articles, of which 27 were selected after an evaluation of their titles and abstracts. Full articles were analyzed, and 19 in-vitro studies and 1 systematic review were considered eligible for the review [Table 1].

## 3. Results

### 3.1. Maintenance of the Preload According to the Type of Connection

Several mechanisms can cause screw loosening and loss of preload. One is the embedment relaxation of mating thread surfaces [8]. Ten percent of initial preload can be lost because of embedment relaxation [21]. New screws and bolts all possess rough-textured thread surfaces as a result of the machining process. When torque is applied, energy is dissipated in the smoothing of the mating surfaces, reducing the elongation of the screw. During loading (i.e., settling), a closer adaptation of the threads will occur because the screw–implant interface experiences micromovement and wear of the contact surfaces. Rough surfaces and large external loads tend to increase this effect and result in greater settling [48]. A second mechanism is a reduction in preload, resulting from either tightening friction or distortion of the screw material [21].

The relationship between applied torque and preload depends on several factors, including screw geometry, material properties, surface texture, degree of lubrication, rate of tightening, and integrity of the joint [49]. Cyclic loading, which simulates functional loading, can significantly influence the overall intimacy of the settling of abutments into implants and their mechanical interlocking at the bone–implant interface [50].

Monitoring of screw torque provides a clearer understanding of the role of the screw and the significance of implant connection design on the maintenance of preload. Several studies have been conducted to resolve this issue. In-vitro static studies have been developed to evaluate torque maintenance in implant–abutment interfaces without the application of any external dynamic forces. In contrast, studies with cyclic loading have been carried out to simulate clinical situations in which implants and prostheses are subject to multiple dynamic/occlusal forces. Torque application studies have been conducted with single and multiple tightening. 

(a) Maintenance of preload after single tightening

Static mechanical behavior after one tightening was evaluated in two studies: Jorge et al. 2013 and Al-Otaibi et al. 2018 [38,44]. A torque of 35 Ncm was applied to the abutment screw in the study of Al-Otaibi et al.; an insertion torque of 20 or 30 Ncm, recommended by the manufacturer, depending on the abutment, was applied by Jorge et al. Retention screws received insertion torque; after 3 min, the initial detorque was measured using a torque gauge by Jorge et al. Al-Otaibi et al. used three protocols in which abutment screws were subjected to different maintenance of torque application (instant, 10 s, 30 s), and the removal torque value (RTV) was recorded with a digital torque gauge. The number of implants included in the studies ranged from 4 to 30. The type of connection examined was the external hexagon implant and Morse taper in Jorge et al., whereas Al-Otaibi et al. (2018) focused on implants with internal connections.

(b) Maintenance of the preload after multiple tightening

Five studies (Cashman et al. (2011), Ferreira et al. (2012), Bernardes et al. (2014), Al-Otaibi et al. (2017), Kim et al. (2020)) [20,35,36,42,47] have investigated the mechanical behavior of different connections as a function of time and tightening and untightening sequences. The studies were designed to test if multiple tightening and untightening sequences of abutment screws influenced the loss of preload. Cashman et al. applied a 35 N abutment torque twice, 10 min apart, in tissue-level implants (internal hexagon), and RTVs were obtained 1 h later. Morse taper implants submitted to retightening and detorque measurements were evaluated by Ferreira et al. The authors applied an insertion torque of 30 Ncm to the retention screw of the tapered abutment; after 3 min, the detorque value was measured 10 times for each specimen. In Bernardes et al., three implant–abutment interfaces were tested (external hexagon, internal hexagon, and internal conical). Each sample was submitted to five tightening/untightening sequences, with a pause of 5 min before the screw was loosened. Al-Oitabi et al. (2017) examined the effect of different torque application techniques on the removal torque of internal connection implants. The torque experiment consisted of torquing the screws to 35 Ncm once, retorquing them once, and retorquing them twice. Kim et al. (2020) evaluated and compared the relationship between the level of applied torque in external and internal hexagon implant–abutment connections and the internal octagon. This study consisted of tightening each abutment in the corresponding implant at a torque of 30 Ncm twice at 10 min intervals.

In these studies, the RTV of the abutment screw was measured after multiple tightening with a digital torque meter (Cashman et al. (2011), Al-Oitabi et al. (2017), Kim et al. (2020)), an analogic torque gauge (Ferreira et al. (2012)), and a screw tightening machine (Bernardes et al. (2014)). The number of implants present in the studies varied from 4 to 50.

(c) Maintenance of the preload after single tightening and the application of cyclical load

Nine studies (Khraisat et al. (2004), Piermatti et al. (2006), Tsuge et al. (2009), Butignon et al. (2013), Jorge et al. (2013), Shin et al. (2014), Gil et al. (2014), Xia et al. (2014), Tsuruta et al. (2018)) [21,29,32,33,38,39,40,41,43] evaluated the load fatigue performance of implant–abutment interface designs. Samples underwent cyclic loads between a minimum of 10 N (Tsuruta et al.) and a maximum of 1450 N (Gil et al.) for 1 × 10^6^ cycles (Khraisat et al., Piermatti et al., Tsuge et al., Jorge et al.), 1 × 10^5^ cycles (Shin et al.), 5 × 10^6^ (Xia et al.), 0.5 × 10^6^ (Butignon et al. and Gil et al.), and 2000 cycles of loading in the study of Tsuge et al. The RTV of the abutment screw was measured after loading (postloading). For this purpose, some of these studies used a torque gauge (Khraisat et al., Tsuge et al., Butignon et al., Jorge et al.) and others used a digital torque meter (Piermatti et al., Park et al., Shin et al.). The loss of preload was investigated in Tsuruta et al. using a torque wrench made by the manufacturer. In the study of Gil et al., no mention is made of the instrument used to evaluate the loss of preload. The number of samples evaluated in all studies varied from 30 to 120. The studies by Khraisat et al. and Butignon et al. were conducted only on external connection implant systems, and Xia et al. only focused on implants with internal connections.

The remaining studies tested specimens with either external or internal implant–abutment connections (Piermatti et al., Tsuge et al., Jorge et al., Gil et al., Shin et al., Tsuruta et al.). Screws were tightened to manufacturers’ recommendations, varying from 20 to 35 Ncm, with the exception of the study of Xia et al., in which implant–abutment assemblies were randomly assigned to three tightening groups (24, 30, and 36 Ncm).

(d) Maintenance of the preload after multiple tightening and the application of cyclical load

Cashman et al. (2011) [35] compared the abutment fatigue resistance to a stimulated function in 40 implants of internal connection using removal torque values as an indication of residual preload, with a digital torque gauge. Application of torque of 35 Ncm was carried out twice, 10 min apart, and postfatigue was obtained 1 h later. Fatigue cycling was carried out using the Bose Electro Force 3300 (Bose Corporation, Eden Prairie, MA) linear electromotor. The applied load was varied sinusoidally at 15 Hz for 5 × 10^6^ cycles between 10 and 200 N. The effect of repeated screw joint closing and opening, after cyclic loading with a chewing simulator (0.5 × 10^6^, 1 Hz, 75 N), was evaluated by Arshad et al. [45] in 30 implants with a hexagonal conical connection. The abutment screw was tightened to 12 and 30 Ncm. RTV measurements were made with an electronic torque meter.

### 3.2. Assessment of Possible Deformations at the Different Interfaces after Repeated Application of the Tightening Torque

Scanning electron microscopy (SEM) is useful to evaluate prosthetic abutment screw surfaces for better interpretation of the effects of tightening/untightening procedures on the surface texture and the plastic deformation of these components.

SEM examination was conducted in seven studies to evaluate the surface changes of the abutment screw thread and the implant hexagon corner after loading.

Two of the studies evaluated the surface changes in the abutment screw and implant connection using SEM, before and after loading (Cashman et al., Arshad et al.) [35,45], and five evaluated the surface topography of implants only after loading [32,33,37,39,41] (Xia et al., Murmura et al., Butignon et al., Khraisat et al., Tsuge et al.). The torque used in the various studies ranged from 12 to 36 Ncm. The number of implants used in the different studies ranged from 30 to 70. Samples underwent cyclic loading between a minimum of 10 N and a maximum of 300 N. The designs of the connections evaluated were external hexagon in the studies of Khraisat et al. and Butignon et al., and internal connection in those of Murmura et al., Cashman et al., Tsuge et al., Arshad et al., and Xia et al.

### 3.3. Evaluate The Sealing Capability Of Different Implant Connections against Microleakage

The presence of gaps at the implant–abutment junction is one of the main factors that contribute to peri-implant inflammation. The gap acts as a microbial colonization site, which may result in loss of supporting bone. Differences in the connection design appear to influence the bacterial leakage at the implant–abutment interface [51]. As the success of implant treatment is based on the ability to maintain osseointegration, it is essential that the implant has a precise fit with the respective abutment [52].

In the systematic review of Mishra et al., (2017) [53], 30 articles were selected: 10 studies were conducted with dynamic loading ranging from 16,000 to 1,200,000 cycles, and the remainder were either conducted without loading or under static loading conditions. The follow-up period of studies ranged from five minutes to five years. Twenty-six studies were conducted using microorganisms: two using dyes, one with deionized water, and one with acrylic resin. The torque used in various studies ranged from 15 to 35 Ncm. The number of implants used in the different studies ranged from 3 to 150. Of these 30 studies, only one was conducted on humans, with a follow up of five years. Almost all studies showed that there was some amount of microleakage at the abutment–implant interface. Microleakage was significantly less in Morse taper implants in comparison to other implant connections. Many studies showed less microleakage in static loading conditions and increased microleakage in dynamic loading conditions. 

Ricomini Filho et al., (2010) [34] evaluated bacterial penetration of the implant–abutment interface of conical and external hexagon connections subjected to thermal cycling and mechanical fatigue (120 N). The assemblies were immersed in tryptic soy + yeast extract broth containing *Streptococcus sanguinis* and incubated at 37 °C and in 10% CO_2_ for 72 h. Detorque values were recorded. The bacterial penetration was assessed, and SEM micrographs of the external hexagon abutment showed no bacterial cells.

Gil et al. (2014) [29] evaluated the microgap size in external and internal connections in a total of 100 samples, with a tightening torque of 45 Ncm in both systems. Five specimens were sectioned along the longitudinal axis to evaluate the microgap size by SEM. Internal connections presented a lower microgap.

In 2019, He et al. [46] developed numerical and experimental methods for investigating the formation of microgaps and the change in contact area at the implant–abutment interface of conical and external hexagon connections under oblique cyclic loading. Abutments were screwed into five implants of each connection with a torque of 20 Ncm. After loading, the samples were scanned using micro-CT, with silver nitrate as a high-contrast penetrant. Ninety percent of the samples of conical connections showed leakage into the internal implant space at a load of around 100 N, while over 80% of those in external hexagons did so at a load of around 40 N.

## 4. Discussion

### 4.1. Maintenance of the Preload According to the Type of Connection

(a) Maintenance of preload after single tightening

The results of Jorge et al. and Al-Otaib et al. [38,44] corroborate those of previous studies, which found that all detorque values were lower than the insertion torque in the baseline in the external hexagon connection and Morse taper (Jorge et al.) and the internal connection (Al-Otaibi et al.). The loss of torque loss a few minutes after torque application is expected and can be explained by a phenomenon known as the sedimentation effect or embedment relaxation [25,54]. This phenomenon assumes that all machined surfaces exhibit a certain degree of microroughness, due to which the surfaces are not perfectly plane. Thus, when the screw receives torque for the first time, contact between the threads occurs; after a few seconds or minutes, the surfaces between the components in the contact area deform and flow. This explains why, clinically, it is recommended to retighten the retaining screw 10 min after the initial torque is applied. According to Breeding et al. [54], the deformation and flow of the components can reduce the torque by 2% to 10% in the first moments after tightening.

Investigation of the effect of different maintenance times of torque application and screw loosening was the aim of the study of Al Otaibi et al. [44] in internal hexagon implants. The mean RTVs were lower than the applied torque for all the protocols. The highest mean RTV was found in the immediate protocol. Maintaining the torque for a prolonged time (10 or 30 s) was not significantly associated with higher preload compared to instant torque application. One possible elucidation in this regard could be that when torque is maintained for a certain time (10 or 30 s), a significant portion of the plastic deformation that occurs mainly during the first few seconds is compensated for, avoiding excessive loss of the detorque value compared to the group submitted to an instant application of torque [55].

(b) Maintenance of the preload after multiple tightening

Because the retorque value measured after screw loosening is an indirect measurement of the remaining preload, the aim of these studies was to evaluate the torque maintenance of the retention screws’ abutment, in different connections, after repeated tightening/loosening cycles of the screws. The torque loss, after multiple tightening, demonstrates that part of the insertion torque used to generate the preload is lost even when no external force is applied to the system. In general, RTVs were found to be lower than tightening torque values. This reduction can be attributed to the phenomenon of the settling effect [56,57,58]. The settling effect occurs because no surface is completely smooth, which causes the presence of high spots on the internal threads of implants and screw threads. These high spots become flattened because they are the only contacting surfaces upon application of the initial tightening torque. Consequently, the torque required to remove a screw is lower than the torque initially used to place it.

Clinically, the current results indicate that the retention screws should be retightened after 3 min of insertion before masticatory loading occurs. In addition, a careful follow-up of the implant-supported prosthesis should be performed because simulated masticatory loading induces screw loosening [36].

In the study of Al-Otaibi [44], removal torque was found to be 79.8% of the applied torque. The results of this study also showed that the retorqued-once application technique resulted in significantly higher RTVs compared to those of the torqued and retorqued-twice techniques. When torque is applied for the first time, some of the torque is used to flatten surface microroughness on the implant’s internal threads and the screw surface. The second application of torque generates the desired preload, and this may explain why the retorqued-once application technique resulted in higher RTVs than the torqued technique [42]. Corroborating these results, the study of Kim et al. (2020) confirmed that it should be taken into consideration that loss of preload due to the settling effect can lead to screw loosening. The mean values of initial removal torque were higher in the internal octagon connection than those of the external connection.

In conflict with these studies, Cashman et al. [35] found no significant difference in RTVs, although the focus of this study was limited to the comparison of internal connection abutments from two manufacturers. The literature reports different preload results because of the use of many different methods for its measurement and evaluation.

The results of Rocha Bernardes et al. [20] did not observe any significant preload change (with titanium screws) after five sequences of tightening/untightening, corroborating the findings of Cashman et al. The samples were used a single time, and no implant was ever reused. This study also found that external hexagon implants showed the lowest preload values generated in the cervical third of the implant, whereas the internal hexagon implants displayed the highest values for preload. Conical implant connections demonstrated stronger structural reinforcement within the internal connections, regardless of whether a torque of 20 or 30 Ncm was applied; however, the latter torque is more appropriate for this implant design. According to this study, a torque of 32 Ncm was mechanically better for Morse taper implants because it did not apparently deform the implant walls more than the deformation caused by a torque of 20 Ncm, whereas it also increased the resistance of the screwed joint to external loads. Screw torque values are also important variables in the retention system of an implant, the stability of which is not determined solely by the interface design or the screw type. Ideal torque amounts for each type of connection should be evaluated. Screw tightening should result in the optimal preload that minimizes screw loosening and fracture [20].

(c) Maintenance of the preload after tightening and the application of cyclical load

Cyclic loading forces during physiological function that do not exceed the maximum strength of an implant–abutment connection may loosen the implant–abutment connection gradually or make it fail due to fatigue. The reason for fatigue failure is either a lack of force-fitting or form-closure of the connection design. The critical reason for the loosening of the implant–abutment connection is the loss of preload at the abutment screw and the resulting unscrewing or fatigue failure of the screw material. RTV has been used as a measurement of preload in numerous studies to evaluate interface stability following fatigue tests [14]. The torque loss may be explained by the fact that the screws are subjected to a mechanical effect known as embedment relaxation, described previously. Because the contacting surface between the screw and the implant cannot be machined to be perfectly smooth, high spots will be the only contacting surfaces when the initial tightening torque is applied. The contacting surface will adapt to smooth the surface, thus leading to preload loss [59].

Study results relating to the maintenance of preload after multiple tightening and application of cyclical load have presented diversity that may be explained by the range of the applied load (from 10 to 1450 N), number of loading cycles (from 2000 to 5 × 10^6^), different fatigue machines, and the number of samples evaluated (from 30 to 120). Some studies compared the different implant designs available, and others included only one kind of connection system.

Many authors indicate that external connection systems present better fatigue behavior due to the differences in force-fit in the connection design [60,61]. In agreement with these findings, we identified the studies of Shin et al. and Gil et al. [29,40]. Regarding fatigue results, Shin et al. showed that the external butt joint was more advantageous than the internal cone in terms of postload removal torque loss. In the study of Gil et al., the external hexagon interface showed superior results compared to the internal hexagon interface. In the study of Jorge et al., after mechanical cycling, a statistically and significantly lower loss of detorque was verified in the Morse taper group compared to the external hexagon group.

Regarding implant design, there was no difference found between the behavior of internal connection and external hexagonal implant systems in the studies of Piermatti et al., Tsuruta et al., and Tsuge et al. [21,33,43]. The results of Piermatti et al. suggest the importance of screw design on the stability of the screw and maintenance of preload. In the study of Tsuruta et al., after 2000 cycles of compressive tensile loadings, RTVs of the abutment screw presented no statistical differences among the three groups (internal, external, and conical connection); however, this study used the fewest loading cycles. Finally, Tsuge et al., revealed that the postloading preload was significantly higher than initial preload in both internal and external connections and indicated that the implant–abutment connection did not have an effect, but the abutment screw material did. Titanium alloy abutment screws were less likely to come loose.

The load application reduced the mean values of the preload significantly in external hexagon connection implants in the studies of Butignon et al. and Khraisat et al. [32.39]. Although there was a significant decrease in the postload reverse torque values in the study of Khraisat et al., screw loosening could not be detected statistically. This may indicate that the remaining tightening torque would serve clinically for a longer period. Similarly, but in the case of an internal connection, the study of Xia et al. [41] revealed that in comparison with the unloaded specimens, the specimens that experienced fatigue loading had decreased RTVs. It was also concluded that fatigue loading would lead to preload loss.

(d) Maintenance of the preload after multiple tightening and the application of cyclical load

In the studies of Cashman et al. and Arshad et al. [35,45], the aim was to investigate if repeated screw joint closing and opening cycles would affect the abutment screw removal torque. 

The results of the study of Arshad et al. indicate that the RTV was considerably lower than the insertion torque in the conical hexagon connection. These results corroborate previous studies, which reported that all screw types display some decay in preload with repeated tightening. The result depends on screw material, intrinsic metallurgic properties of the raw material, and the manufacturing process. These factors could explain the variations observed by Arshad et al. in the torque values between samples of the same group. Previous studies have shown that not only screws from different manufacturers but also screws from different lots of the same manufacturer could lead to different maximum preload torque before fracture [22,62]. Clinically speaking, increasing the number of times an abutment screw is closed and opened will eventually result in the reduction of removal torque and an increased risk of screw loosening. Arshad et al. also observed, in conical hexagon internal connections, that using a new screw could not significantly increase the value of removal torque and that restricting the amount of screw tightening was more important than replacing the screw.

Cashman et al., did not determine a significant loss of RTV postfatigue loading despite similar test parameters. The purpose of the study of Cashman et al. was to compare the abutment fatigue resistance to a simulated function in a specific brand control abutment relative to a third-party-compatible abutment. The differences in chemical composition, manufacturing, and surface treatment indicate a need for independent verification of functional compatibility. Different abutment manufacturers result in a difference in RTV postfatigue loading. The control abutment demonstrated a greater RTV than the third-party-manufactured component.

### 4.2. Assessment of Possible Deformations at the Different Interfaces after Repeated Application of the Tightening Torque

Scanning electron microscopy (SEM) was carried out to determine the characteristics of the interface microgap, compare thread geometry, and evaluate surface characteristics between systems.

SEM examination was conducted by Khraisat et al. (external hex implants) and Tsuge et al. (internal and external implants) [32,35]. These studies evaluated the surface changes of the abutment screw thread and the implant hexagon corner, before and after loading, with 1 × 10^6^ cycles (Khraisat et al.) and 2000 cycles (Tsuge et al.). In the study of Khraisat et al., mild burnishing and scuffing of the abutment screw thread surfaces were observed, after tightening, in control specimens that were not loaded. Marked burnishing was observed at the hexagon corners on the compression sides.

In the study of Tsuge et al., damage was observed on the threads of the abutment screws and the screw surfaces (roughening, stemming) on the upper and lower flanks, which was probably due to screw tightening. However, no abnormal wear or damage due to micromovement or bending caused by cyclic loading was observed on the abutment screws in any of the samples.

SEM was also carried out in the study of Cashman et al. [35] after 5 × 10^6^ cycles of loading to compare thread geometry and evaluate surface characteristics in internal connections. Differences in surface finish were visualized in postfatigue cycling, such as ductile delamination and rough surfaces in the profiles of the threads. Visual differences at the macro/microscopic level were also apparent in the thread geometry, with third-party abutments demonstrating considerably greater variation in geometrical architecture than control specimens.

In the study of Xia et al. [41], the dynamic fatigue performance (5 × 10^6^) of implant–abutment assemblies with internal connections and different tightening torque values was investigated. Under-tightened implant–abutment assemblies (24 Ncm) failed to survive fatigue tests (crack propagation), whereas implant assemblies in the recommended and over-tightened torque groups (30 and 36 Ncm, respectively) had intact implant–abutment interfaces, as proven by SEM.

The surface topography of one screw in each group, before and after cyclic loading (0.5 × 10^6^), was evaluated by Arshad et al. [45] and compared with an unused screw. SEM analysis after loading displayed destruction of the thread abutment screw surface (desquamation and destruction of the superficial layer). In general, it could also be seen that even a precisely machined new screw was not highly smoothed.

A single study contradicts all of these findings: Murmura et al. [37] used SEM and demonstrated the absence of gaps or mechanical deformations at the stump’s closing edge on its implant after the application of the cyclic load (1 × 10^6^) in internal hexagon and internal octagon connections.

### 4.3. Evaluate the Sealing Capability of Different Implant Connections against Microleakage 

In the systematic review of Mishra et al. (2017) [53], a maximum study showed that there was some amount of microleakage at the abutment implant interface. External hexagon implants failed to completely prevent microleakage in both static and dynamic loading conditions of implants. Internal hexagon implants, particularly internal conical (Morse taper) implants, are highly promising in the case of static loading and showed less microleakage in dynamic loading conditions. Torque values recommended by the manufacturer should be strictly followed to achieve a better seal at the abutment–implant interface. Zirconia abutments are more prone to microleakage than titanium abutments, and their use should be discouraged. Zirconia abutments should only be restricted to cases where there is a high demand for aesthetics. These results corroborate the study of He et al. [46] (2019) in which the conical connection showed more resistance against the formation of microgaps at the implant–abutment interface than the external hexagonal connection. Additionally, Gil et al. [28] concluded that internal connections had a smaller microgap than external connections, with significant statistical differences. Very good adaptation between the implant and the screw-retained abutment was observed; in many cases, the distances were smaller than the bacteria diameter, thus preventing infiltration of microorganisms. In contrast, Ricomini Filho et al. [34] observed a better bacterial seal in the group with an external hexagon with a universal post than in groups with conical connections. These authors found that the external hexagon connection could have acted as a physical barrier, blocking bacterial penetration toward the inner part of the implant. SEM micrographs show no bacterial cells on the surface of the external hexagon abutment screw, thus confirming the microbiological assay. The methodology of rubbing a paper point on the inner part of the implant was probably unable to assess the microbial colonization on the implant platform, justifying the need for future studies to confirm these findings.

In vitro investigations showed that a major portion of conical connection systems presents a microgap under static forces smaller than 10 μm [63], demonstrating a better fit of the abutment into the fixture but not eliminating it completely. Other authors have shown minimal abutment movement and microgap formation under axial and oblique forces but good resistance to torque loss and screw loosening [64]. Internal cone implants have interface force transfer characteristics similar to those of a one-piece implant, but an absolute bacterial seal cannot be achieved in a two-piece implant system. For these reasons, conical abutment should be preferred to other connection systems to minimize bacterial microleakage [65]. Corroborating these findings, Gherlone et al. [66] tested, in an in-vitro study, a new internal conical connection design characterized by a double-taper principle. The authors evaluated and compared a new connection design, named double-action tight (DAT), with other internal connections. To investigate bacterial microleakage, the inner part of each system was inoculated with an *Escherichia coli* suspension. They found that in the DAT connection group, 7 of 10 total implants showed no bacterial infiltration at 96 h. This new internal conical design should reduce bacterial infiltration by constructing a physically tight connection with a high level of precision in the submicrometer range. Additional studies are necessary to better understand the stability of this new type of internal connection over a longer period, with different bacteria and subject to the mastication function.

## 5. Conclusions

This review found that different studies have been performed using a variety of approaches, thus often making the studies difficult to compare. As a result, it is difficult to draw conclusions about which abutment system behavior is optimal.

Considering the proposed objectives, we can draw the following conclusions:

1. Maintenance of the preload: Internal connections have a higher preload value than that of the external hexagon design. The conical configuration can spread the load along the fixture and the surrounding bone more homogeneously than both the external hexagon and traditional internal connections.
2. Assessment of possible deformations at different interfaces after repeated application of tightening torque: Damage was observed in the threads of the abutment screws, before and after loading, in external and internal implant–abutment connections.
3. Evaluation of the sealing capability of different implant connections against microleakage: All connections presented some microgaps and bacterial microleakage. However, the performance of the conical connection systems appeared to be superior to that of other systems.

Further in-vivo prospective studies are needed to build evidence of the best-performing connection over the long-term while bearing in mind the other factors that can affect clinical results.

## Figures and Tables

**Table 1:** Summary of included articles.

Author Year Type of Study	Purpose	Type of Connection Number of Implants Torque Used	Intervention	Evaluation	Outcome of the Study
Khraisat A et al. [32] 2004 In Vitro	Effect of lateral cyclic loading with different load positions on abutment screw loosening	EXTERNAL HEXAGON (Branemark) divided into three groups (*n* = 50) 32 Ncm	A-Cyclic load 50 N 1 × 10^6^ cycles applied centrally. B- cyclic load 50 N 1 × 10^6^ cycles applied eccentrically. C-control unloaded.	Torque gauge Micrometer microscope (1 μm) SEM	RTVs were preserved under eccentric lateral loading compared with centric loading. SEM analysis revealed mild burnishing and scuffing of the screw thread surfaces in all groups. Marked burnishing was observed at the hexagon corners on the compression sides for group B implants.
Piermatti J et al. [21] 2006 In Vitro	Examine effects of connection design upon screw stability	EXTERNAL HEXAGON (10 BioLok; 10 Nobel Biocare) INTERNAL CONNECTION (10 Zimmer; 10 Astra Tech) (*n* = 40) 32 Ncm	10 samples of each system were loaded to 200 N for 1 × 10^6^ cycles. Screws were tightened to manufacturers’ recommendations. Torque audits were done after 250,000, 500,000, 750,000, and 1,000,000 cycles.	Torque meter	The design of the joint (i.e., internal vs. external connections) was not a significant factor in loss of torque. What did appear important was the screw design. Bio-Lok abutment screws (external connection) maintained their tightness in the best of the four systems.
Tsuge T et al. [33] 2009 In Vitro	Evaluate the effect of eccentric cyclic loading on abutment screw loosening in internal and external hexagon implants with two screw materials	INTERNAL HEXAGON EXTERNAL HEXAGON (*n* = 64) 4 groups20 Ncm	Load was applied one million times (1.0 × 10^6^ cycles] RTV measured before (initial preload) and after loading (post-loading). Changes in the superstructure and movement of the abutment were inspected using visual and tactile checks every 50,000 cycles. Damage to the surfaces of selected abutment screws was observed using SEM.	Torque gauge Fatigue testing machine SEM	In all the groups, postloading preload was significantly higher than the initial preload. Implant–abutment connection did not have an effect, but the abutment screw material did. Screws damage was probably due to screw tightening and was observed on the flank near the crest. However, no abnormal wear or damage due to micromovement or bending caused by cyclic loading was observed on the abutment screws in all the groups.
Ricomini Filho AP et al. [34] 2010 In Vitro	Evaluate the preload loss and bacterial penetration through the implant–abutment interface of conical and external hexagon connection systems subjected to thermal cycling and mechanical fatigue.	EXTERNAL HEXAGON MORSE TAPER (*n* = 6) 32 Ncm 15 Ncm	The assemblies were subjected to a thermal cycling regimen (1000 cycles of 5 and 55 °C) and to mechanical fatigue (1.0 million cycles, 1.0 Hz, 120 N). The assemblies were immersed in tryptic soy + yeast extract broth containing *S sanguinis* and incubated at 37 °C and 10% CO_2_ for 72 h. Detorque values were recorded. The bacterial penetration was assessed, and the abutments were observed by SEM.	Electronic torque controller Mechanical fatigue machineSEM	All screw abutment systems showed significantly higher detorque values when subjected to TM, and all conical systems presented bacterial penetration. The results show no relationship between the preload loss and bacterial penetration. SEM micrographs show no bacterial cells on the surface of the external hexagon abutment screw, confirming the microbiological assay.
Cashman PM et al. [35] 2011 In Vitro	Compare the abutments fatigue resistance to simulated function, using Removal torque Values as an indication of residual preload at the implant–abutment interface.	INTERNAL HEXAGON (Straumann) (*n* = 40) Four groups: 10 abutments from each manufacturer were evaluated for RTV with and without fatigue loading 35 Ncm	Baseline: torque was carried out twice, 10 min apart. RTV obtained 1 h later. Postfatigue: torque was carried out twice, 10 min apart. RTV postfatigue obtained 1 h later. 10 to 200 N, 15 Hz for 5 × 10^6^ cyclesSEM was carried out at baseline and post fatigue cycling to visualize thread geometry and abutment–implant interface.	Digital torque gauge Moving-magnet linear motor to load specimens SEM	The effect of component manufacturer resulted in a significantly higher RTV in the control group, indicating greater residual preload. There was no significant decrease in postfatigue RTV for either manufacturer compared to baseline. Differences in surface finish and machining tolerances were visualized with SEM.
Ferreira MB et al. [36] 2012 In Vitro	Evaluate the torque maintenance of retention screws of tapered abutments and cylinders of Morse taper implants submitted to retightening and detorque measurements	MORSE TAPER (*n* = 12) 30 Ncm	Detorque values were measured by an analogic torque gauge after 3 min of torque insertion. The detorque was measured 10 times for each retention screw of groups I and II, for a total of 120 detorque measurements in each group.	Analogic torque gauge	The abutment and cylinder screws exhibited torque loss after insertion, which indicates the need for retightening during the function of the implant-supported prostheses.
MurmuraG et al. [37] 2013 In Vitro	Evaluation of the preload distribution in screw-retained implant systems under cyclic load.	INTERNAL HEXAGON (35 Xsign) INTERNAL OCTAGON (35 SSO) (*n* = 70) 25 Ncm 35 Ncm	Cyclic load between 20 and 200 N for 1 × 10^6^ cycles. After mechanical tests, samples were sectioned along the long axis and analyzed under SEM. Five implants with internal hexagon and five implants with internal octagon were collected for photoelastic analysis.	The loss of preload was evaluated through the presence or absence of abutment mobility SEMPhotoelastic analysis	The design of the abutment connection area affects the functional load transfer to the fixture and connection screw; screw-retained abutment based on an internal octagonal connection is less likely to come loose after cyclic load.
Jorge VA et al. [38] 2013 In Vitro	Evaluate the role of the implant/abutment system on torque maintenance of titanium retention screws and vertical misfit of screw-retained implant-supported crowns before and after mechanical cycling.	MORSE TAPER EXTERNAL HEXAGON (*n* = 30) 20 Ncm 30 Ncm	Retention screws received insertion torque and after 3 min, initial detorque was measured. Crowns were retightened and submitted to cyclic loading under 30 degrees of 130 ± 10 N, 2 Hz, 1 × 10^6^ cycles. Final detorque was measured and vertical misfit using a stereomicroscope.	Stereomicroscope Torque gauge Electromechanical equipment for mastication fatigue	All groups presented a significant decrease in torque before and after mechanical cycling. The Morse taper connection promoted the highest torque maintenance. Mechanical cycling reduced the vertical misfit of all groups.
Butignon LE et al. [39] 2013 In Vitro	Evaluate the effectiveness of 3 types of abutments in the maintenance of screw joint preload before and after cyclic loading and observe possible microdamage in the structure.	EXTERNAL HEXAGON (Neodent) (*n* = 45) 1. machined titanium (Ti) abutment 2. premachined gold (Au] abutments 3. machined zirconia (ZrO2) abutments 20 Ncm; 32 Ncm	Static vending test using five specimens of each group was conducted to determine load applied in the cyclic loading test. Ten specimens of each group measured the RTV. A cyclic loading (0.5 × 10^6^ cycles, 15 Hz) between 11 and 211 N, angle 30°, was applied. Postload RTV was measured after cyclic loading. SEM was used to detect possible microdamage in the structure of components.	Torque gauge Fatigue test machine SEM	The load application reduced the preload means significantly in all groups. SEM images showed evident structural changes in the mating surface of abutments, which are related to the loss of retained preload in all groups.
Gil FJ et al. [29] 2014 In Vitro	Evaluate microgap size and fatigue behavior of external and internal connections.	INTERNAL HEXAGON EXTERNAL HEXAGON (Klockner SK2 and Essential) (*n* = 100) 45 Ncm	Each specimen was sectioned along the longitudinal axis in a total of three slides for SEM.The aim was to find the level of stress at which the sample supported 5 million cycles at 1450 N (15 Hz), which will be considered the fatigue limit.	SEM Servo-hydraulic testing machine	Internal connection presented lower microgap. Very good adaptation between the implant and the screw-retained abutment, preventing infiltration of microorganisms. The fatigue behavior of the external hexagon interface showed superior results compared to internal hexagon interfaces due to better load distribution.
Shin HM et al. [40] 2014 In Vitro	Evaluate the influence of the implant–abutment connection design and diameter on the screw joint stability	EXTERNAL HEXAGON INTERNAL CONE (*n* = 35) 30 Ncm	The initial and postload RTV were measured after 100,000 cycles of a 150 N and 10 Hz cyclic load. The rates of the initial and postload removal torque losses were calculated to evaluate the effect of the joint connection design and diameter on the screw joint stability.	Digital torque gauge	External butt joint was more advantageous than the internal cone in terms of the postload removal torque loss. A wide diameter was more advantageous in terms of the torque loss rate.
Bernardes SR et al. [20] 2014 In Vitro	Determine whether abutment screw tightening and untightening influenced loss of preload in 3 different implant/abutment interfaces or on the implant body	EXTERNAL HEXAGON INTERNAL HEXAGON INTERNAL CONICAL (Neodent) (*n* = 25) 20 Ncm 32 Ncm	Each sample was submitted to five tightening/untightening sequences, with a pause of 5 min after tightening before the screw was loosened. This resulted in 25 tightening/untightening sequences of each design.	Custom benchtop screw-tightening machine (Prosthetic Dept, UCL, Eastman Dental Institute)	There was no immediate significant loss of preload after screw tightening. Tightening/untightening sequences did not result in any significant loss of preload. Conical implant connections demonstrated greater structural reinforcement within the internal connection.
Xia D et al. [41] 2014 In Vitro	Evaluate the dynamic fatigue performance of implant–abutment assemblies with different tightening torque values.	INTERNAL CONNECTION (Zimmer Biomet Dental) (*n* = 30) Three tightening groups: 24, 30, 36 Ncm.	Five specimens of each group were unscrewed and RTV recorded. Another five specimens were subjected to load between 30 and 300 N, 15 Hz for 5 × 10^6^ cycles. RTV postfatigue was recorded if available. Surfaces of specimens were observed with SEM.	Digital torque meter Fatigue testing machine SEM	The specimens that went through fatigue loading had decreased RTV. Insufficient torque will lead to poor fatigue performance of dental-abutment assemblies. Screws should be tightened to the torque recommended by the manufacturer.
Al-Otabi HN et al. [42] 2017 In Vitro	Examine the effect of different torque application techniques on the removal torque of implant-supported fixed complete dental prostheses.	INTERNAL CONNECTION (Nobel Biocare-Replace) (*n* = 4) 35 Ncm	The torque experiment consisted of three protocols: 1. Torqueing screws to 35 Ncm once. 2. Torqueing the screws to 35 Ncm and then immediately retorquing the same screws to the same value. 3. Torqueing the same screws to 35 Ncm three consecutive times. Removal torque was recorded.	Digital torque meter	Retightening abutment screws once after the initial torquing could enhance the removal torque of the screw. Care must be taken when retorquing the screws more than once because this may inversely affect the removal torque.
Tsuruta K et al. [43] 2018 In Vitro	Evaluate three kinds of connection systems from the point of view of microleakage from the gap between the implant and the abutment.	EXTERNAL CONNECTION INTERNAL CONICAL CONNECTION INTERNAL PARALLEL CONNECTION (Nobel Biocare)(*n* = 21) 35 Ncm	Dye leakage was observed from the abutment screw hole to the outside through the microgap under the excessive compressive and tensile load. Each cycle, one compressive load and one tensile load (10 N each), was applied per 1 s and 2000 cycles of loading were performed. Every 500 cycles, the amount of microleakage was statistically compared. After the completion of 2000-cycle loading, RTV of the abutment screw was measured.	Torque wrench (Nobel Biocare) Universal test machine Spectrophotometer	The amount of microleakage from implant–abutment interface was smaller in conical connection than in internal parallel connection. The increase in the amount of microleakage was observed in all three groups. Removal toque of abutment screw after the cyclic loading showed no statistically significant difference among the groups.
Al-Otaibi HN et al. [44] 2018 In Vitro	Examine the effect of different maintenance time of torque application on detorque values of implant abutment screw.	INTERNAL HEXAGON (*n* = 4) 35 Ncm	The abutment screws were subjected to different maintenance time of torque application Protocol A: 35 Ncm “instant” torque application. Protocol B: 35 Ncm torque maintained for 10 s. Protocol C: 35 Ncm torque maintained for 30 s. The procedure was repeated for each protocol five times, in which new sets of screws were used, with a total of 60 new screws.	Digital torque meter	The application of 35 Ncm for different maintenance times of torque application did not appear to affect the detorque value. Maintaining the torque for a prolonged time (10 s or 30 s) was not significantly associated with a higher preload than instant torque application.
Arshad M et al. [45] 2018 In Vitro	Evaluate the effect of repeated screw joint closing and opening cycles and cycling loading on abutment screw removal torque and screw thread morphology.	INTERNAL CONICAL CONNECTION (Dentium) (*n* = 30) Three groups 12 and 30 Ncm	Abutments screw were tightened (12 Ncm), removed, and RTV-recorded. This sequence was repeated five times for G1 and 15 times for G2 and G3. The same screws for G1 and G2 and new screws for G3 were tightened to 12 Ncm; this was also followed by screw tightening to 30 Ncm and retightening to 30 Ncm, 15 min later. RTVs were taken after cyclic loading 0.5 × 10^6^, 1 Hz 75 N. Surface topography of one screw in each group was evaluated with SEM and compared with an unused screw.	Electronic torque meter Chewing simulator SD mechatronic SEM	Using a new screw did not significantly increase the value of removal torque. Restricting the amount of screw tightening was more important than replacing the screw. Torque loss values after loading were not shown to be significantly different from each other.
He Y et al. [46] 2019 In Vitro	Investigate the formation of microgaps and the change in the contact area at the implant–abutment interface of two different connection designs under oblique cyclic loading.	CONICAL CONNECTION EXTERNAL HEXAGON CONNECTION (*n* = 10)20 Ncm	After loading, the samples were scanned using micro-CT, with silver nitrate as a high contrast penetrant, and the level of leakage was assessed.Three-dimensional finite element (FE) analyses were conducted to reveal the microgap formation process.	Micro CT with silver nitrate Fatigue machine	The conical connection showed more resistance against the formation of microgaps at the implant–abutment interface than the external hexagonal connection, although the minimum load required to bridge the internal implant space was within the range of human biting force.
Kim KS et al. [47] 2020 In Vitro	Examine the settling of abutments into implants and the removal torque value under static loading.	EXTERNAL CONNECTION INTERNAL HEXAGON INTERNAL OCTAGON (*n*= 50) 30 Ncm	Ten implant–abutment assemblies were loaded vertically downward with a 700 N load cell at a displacement rate of 1 mm/min. The settling of the abutment was obtained from the change in the total length of the entire implant–abutment unit using an electronic digital micrometer. The postloading RTV was compared to the initial torque value.	Universal testing machine electronic digital micrometer Digital torque gauge	The loss of the preload due to the settling effect can lead to screw loosening during a clinical procedure in the molar region, where masticatory force is relatively greater.
